# Assessment of aortic and peripheral arterial stiffness in patients with knee osteoarthritis by ultrasound Doppler derived pulse wave velocity

**DOI:** 10.1038/s41598-024-52097-1

**Published:** 2024-01-16

**Authors:** Yong Yang, Xiao Liang, Hu Luo, Yu-xin Cheng, Yan Guo, Peng Wu, Yan-li Huang, Jin-peng Zhang, Zhen Wang

**Affiliations:** 1 Department of Ultrasound Medicine, Tangdu Hospital, Fourth Military Medical University, 569# Xinsi Rd, Xi’an, 710038 China; 2 Department of Osteology, Qinhuai Medical Section of General Hospital of Eastern Theater Command, Nanjing, 210000 China; 3 Department of OsteologyTangdu Hospital, Fourth Military Medical University, Xi’an, 710038 China; 4 Department of Ultrasound Diagnostics, Qinhuai Medical Section of General Hospital of Eastern Theater Command, Nanjing, 210000 China; 5 Department of Medical Imaging, Hospital of Unit 96608 of the People′s Liberation Army (PLA), 7# Dongchang Rd, Hanzhong, 723100 China

**Keywords:** Biomarkers, Cardiovascular diseases, Rheumatic diseases

## Abstract

Information regarding regional arterial stiffness assessment in osteoarthritis (OA) was scarce and sometimes contradictory. We aimed to investigate the aortic, lower limb peripheral arterial stiffness and their associations with knee OA. Patients with primary knee OA and matched non-OA controls were prospectively enrolled from two medical centers in China. The carotid-femoral pulse wave velocity (cfPWV) and femoral-ankle pulse wave velocity (faPWV) were measured using a novel ultrasound technique. A total of 238 participants (including 128 patients with knee OA and 110 controls) were included. In OA patients, cfPWV was significantly higher than that of non-OA controls (9.40 ± 1.92 vs 8.25 ± 1.26 m/s, *P* < 0.0001). However, faPWV measurements in OA patients (12.10 ± 2.09 m/s) showed no significant difference compared with that of the controls (11.67 ± 2.52 m/s, *P* = 0.130). Multiple regression analysis revealed that cfPWV was independently associated with knee OA (*P* < 0.0001) after adjusting for the confounding factors including age, gender, smoking, mean blood pressure, body mass index, heart rate, high-sensitivity C-reactive protein and lipids profiles. In contrast, faPWV did not show independent association with knee OA (*P* = 0.372) when after adjusting for confounding factors. In addition, Spearman’s correlation analysis showed cfPWV had a significant correlation with Kellgren-Lawrence score (r_s_ = 0.2333, *P* = 0.008), but no correlation was founded between faPWV with Kellgren-Lawrence score (r_s_ = 0.1624, *P* = 0.067) in OA patients. This study demonstrated that stiffening of aorta, but not lower limb arteries, was independently associated with knee OA. Our findings may call for further implementation of routine aortic stiffness assessments so as to evaluate cardiovascular risk in patients with OA.

## Introduction

Osteoarthritis (OA), a highly prevalent rheumatic musculoskeletal disorder, is the leading cause of disability in ageing population worldwide. Accumulating evidence has revealed that patients with OA have experienced excess cardiovascular diseases (CVD)^1–3^. Common risk factors such as chronic inflammatory, physical inactivity, aging and obesity may contribute to the association between OA and CVD, however, this association cannot be accounted by common risk factors alone^4^. Being one of the earliest detectable adverse manifestations within the vessel wall, arterial stiffness (AS) has been a well-accepted robust independent predictor for CVD and mortality^5–7^. Regional pulse wave velocity (PWV) has been used for reliably assessing AS in practice. Regional PWV is calculated as the pulse wave transit distance divided by the corresponding transit time, reflecting the stiffness of the corresponding arterial segment(s). For example, carotid-femoral PWV (cfPWV), the “gold standard” measurement of AS, mainly reflects the stiffness of the descending, abdominal aorta as well as the iliac artery, whereas femoral-ankle PWV (faPWV) mainly covers the lower limb peripheral arteries.

AS and knee OA share some risk factors and may be interrelated. Furthermore, different segmental AS may have different roles in CVD pathogenesis which often occurs in OA. However, data regarding the regional PWV in OA are scarce and sometimes contradictory^8,9^. Whether and to which extent stiffening of aorta and lower limb arteries are associated with knee OA is unclear. Therefore, here we performed a two-centered prospective cross-sectional case control study to investigate the associations between aortic stiffness (by measuring cfPWV), lower limb AS (by measuring faPWV) and knee OA.

## Methods

### Subjects

In this study, patients with knee OA and non-OA controls were enrolled from the osteology departments of Center 1 (Tangdu Hospital of Fourth Military Medical University, Xi’an, China) and Center 2 (Qinhuai Medical Section of General Hospital of Eastern Theater Command, Nanjing, China), during May. 2021 to Oct. 2022. Knee OA was diagnosed according to the American College of Rheumatology criteria^10^.

The inclusion criteria were primary OA patients with only knee joint(s) involved, with stable hemodynamic conditions, age ≤ 80 years, without antihypertensive or anti-dyslipidemia drugs usage. Here the stable hemodynamic conditions meant the included subject or patient showed stable blood flow velocity spectra when measuring PWV using ultrasound Doppler technique. The non-OA controls were comparable in age and gender with OA patients and were included from the two centers at the same period.

The exclusion criteria were as follows: secondary OA caused by previous knee surgery/trauma, diabetes (treated or untreated), history of coronary artery disease (e.g., chronic stable angina, unstable angina, myocardial infarction), daily nonsteroidal anti-inflammatory drugs (NSAIDs) use ≥ 1 year, arrhythmia (recognized and diagnosed by the ECG signals shown in the ultrasound system used in this study), reduced left ventricular ejection fraction (< 50%, measured by echocardiography based on Teichholz formula), chronic respiratory and/or renal diseases (chronic obstructive pulmonary disease, pulmonary fibrosis, tuberculosis, nephritis, renal failure, etc.), fever, malignancy, other rheumatic disorders (e.g., systemic lupus erythematosus, rheumatoid arthritis, scleroderma, vasculitis, dermatomyositis, spondyloarthritis), severe aortic valvular stenosis or artery lumen stenosis (which led to an abnormal Doppler blood flow spectrum occurred in common carotid, femoral or posterior tibial artery).

This study was approved by local institutional ethical boards of Center 1 (No. 201909-01) and Center 2 (No. DZQH-KYLLFS-21-04). All study protocols were conformed to the principles of the Declaration of Helsinki. Written informed consent was obtained from each participant after study explanation.

### Medical assessment

At the enrollment, the information about demographic characteristics, underlying diseases, medications of the eligible subjects were collected. In patients with OA, the knee, hip and hand joints were examined by X-ray and the control group only had knee, hip joints X-ray. Based on the X-ray imaging, the severity of knee OA was further graded using Kellgren-Lawrence scoring system^11^, where 0 = no changes; 1 = doubtful joint space narrowing; 2 = definite osteophytes and doubtful joint space narrowing; 3 = definite osteophytes, joint space narrowing, sclerosis and possible deformity; and 4 = marked joint space narrowing, large osteophytes, severe sclerosis and definite bone deformity.

The above information collection, knee OA diagnosis and scoring were performed by two osteologists from Center 1 (both with more than 10 years of experiences in OA) and two osteologists from Center 2 (one with 8 years and another 4 years of experiences in OA) and the decisions were reached in consensus. They were all blinded to measurements of PWV.

### PWV measurements

In this study, we measured faPWV and cfPWV of each subject in a quiet, temperature-controlled (22 ± 1 ℃) room, using a same ultrasound unit (G55, VINNO Technology, Suzhou, China) with a 10–12 MHz linear array transducer. This ultrasound device was equipped with an semi-automatic system for measuring regional PWV, which was previously validated to be an accurate and reproducible method^12^. Intra- and inter-observer intraclass correlation coefficients of this method for measuring cfPWV were reported excellent as 0.968 and 0.903^12^. In this study, the PWV measurements were performed by two sonographers from Center 1 and Center 2, with 12 years and 7 years of experiences in echocardiography, respectively. The sonographers were blinded to subjects’ group and the Kellgren-Lawrence scores.

**Figure 1:**
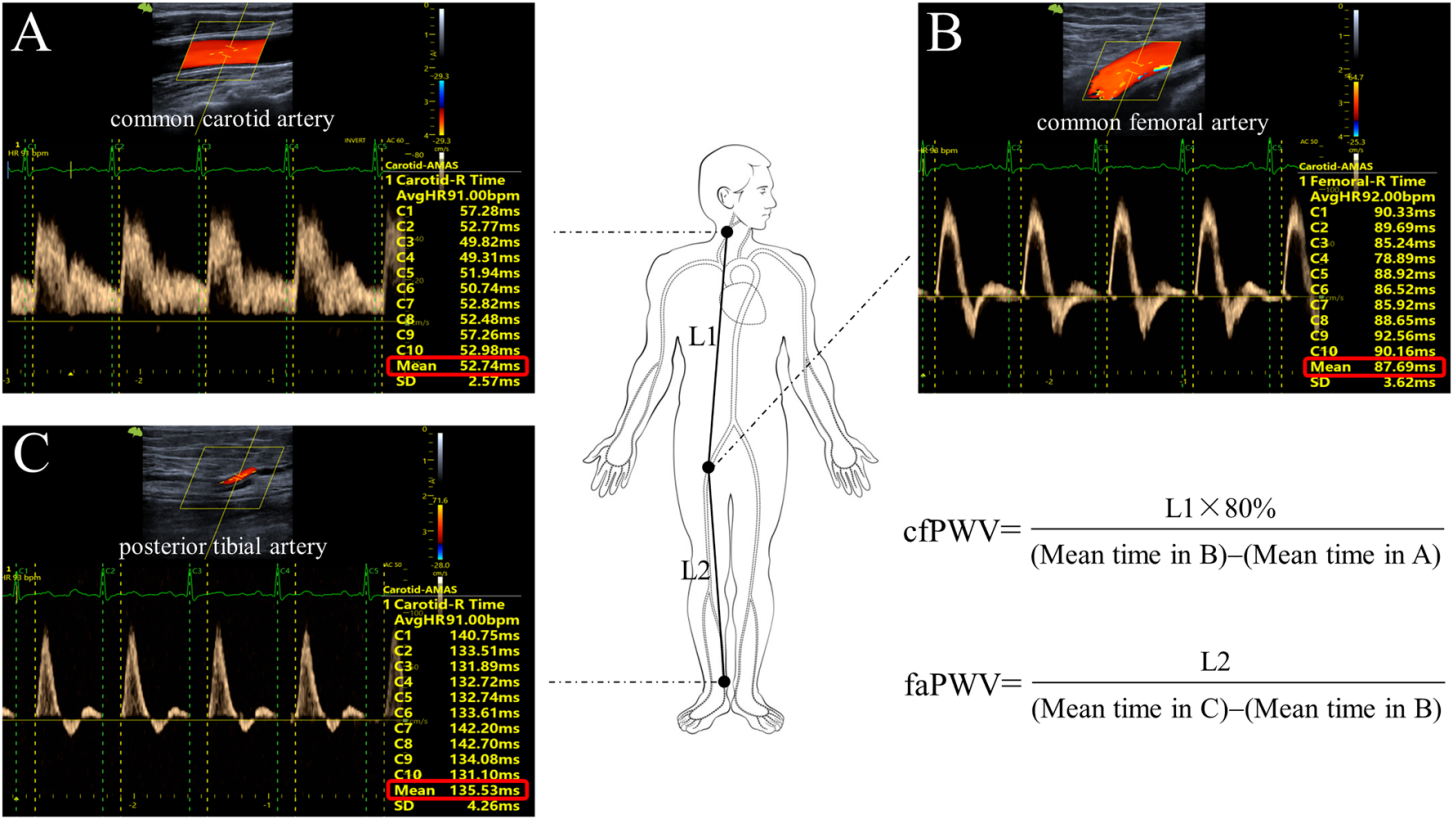
Methodology of measuring regional PWV. The pulse-wave Doppler flow velocity spectrum was recorded at the right common carotid artery (**A**), common femoral artery (**B**) and posterior tibial artery (**C**), respectively. Based on the spectrum, the time interval between peak R wave of ECG and the wave foot was automatically measured from 10 continuous cardiac cycles (for details in Ref.^12^) and the results including the mean value (red box) were shown. L1 and L2 represent the direct straight distances between the sampling points (marked on the body surface) and were averaged from three measurements.

Each subject adopted a supine position at least 5 min rest to achieve hemodynamic stability. Blood pressure (BP) was measured at the right brachial artery using a sphygmomanometer and three measurements were averaged. Then, cfPWV and faPWV were measured using a novel methodology which was described previously^12^. As illustrated in Fig. 1, the transit distance was determined based on the tape measured direct straight distances on body surface. The corresponding transit time can be automatically measured on the Doppler flow spectra with ECG gating. Therefore, PWV was calculated as transit distance divided by the corresponding transit time^13^.

### Laboratory profiles

Lipid profiles, blood glucose, high-sensitivity C-reactive protein (hs-CRP) of the subjects were measured using standard laboratory methods in each center by automated analyzers.

### Statistics

Normal distribution of data was analyzed by Kolmogorov–Smirnov test and was expressed as the mean ± SD. Non-normally distributed data and ordinal data were expressed as median with inter-quartile range (IQR). Unpaired *t*-test was used for the comparisons of parameters with normal distribution between two groups and Mann–Whitney U test was used when in parameters without normal distribution. Categorical data was expressed as number (percentage) and compared by Chi-square χ^2^ test or Fisher’s exact test. Pearson’s correlation or Spearman’s correlation was used to analyze the univariate correlation between possible confounding factors with PWV measurements. Further, multiple regression analysis was adopted to assess the independent associations of aortic or peripheral PWV with knee OA. Statistical software IBM SPSS Statistics version 21 (IBM Corp., NY, USA) was used and a two-sided *P* value < 0.05 was regarded as statistically significant.

### Ethics approval and consent to participate

This study was approved by local institutional ethical boards of Center 1 (No.201909-01) and Center 2 (No. DZQH-KYLLFS-21-04). All study protocols were conformed to the principles of the Declaration of Helsinki. Written informed consent was obtained from each participant after study explanation.

## Results

**Figure 2:**
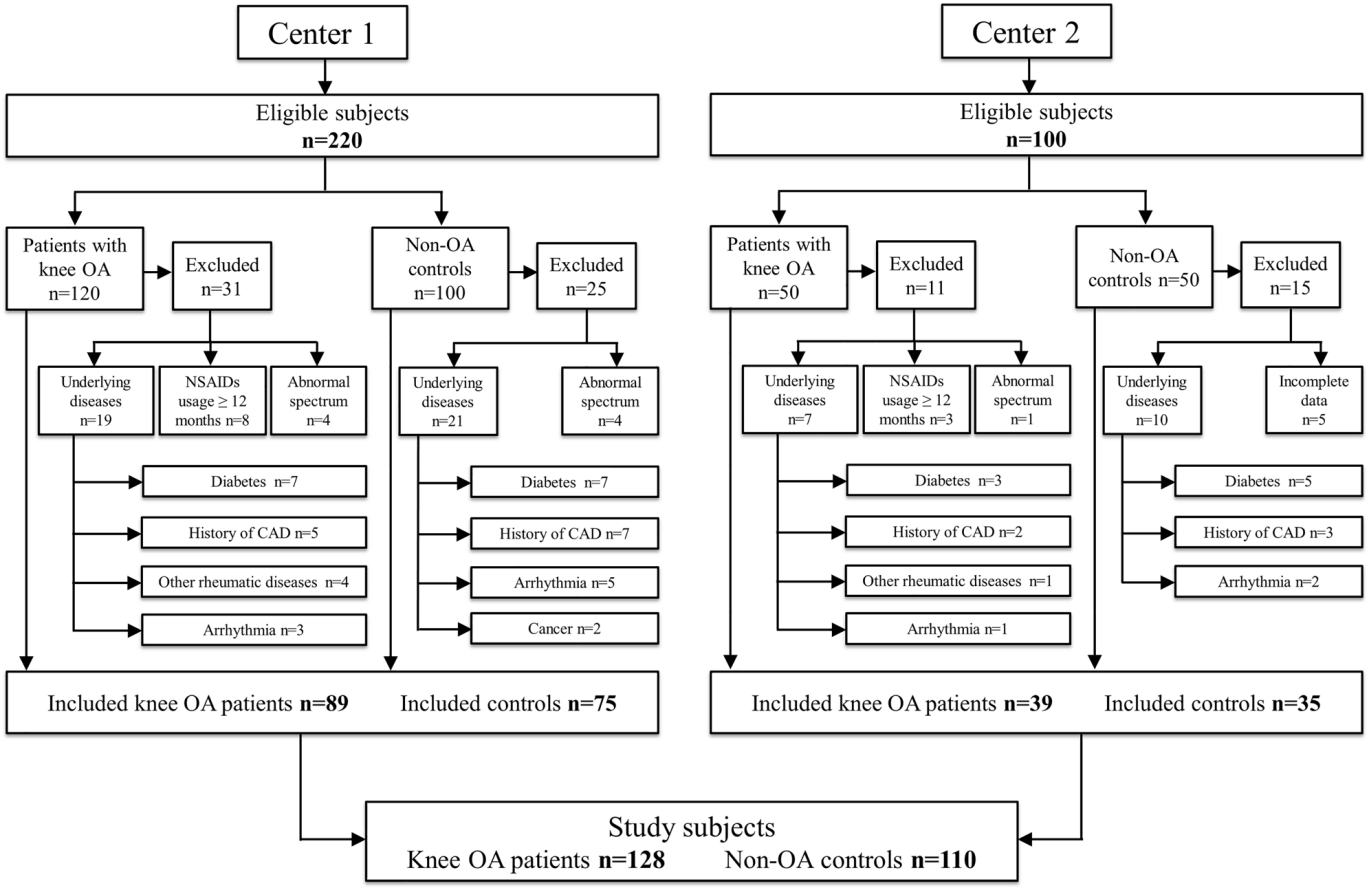
Flowchart of this study. *OA* osteoarthritis, *NSAIDs* nonsteroidal anti-inflammatory drugs, *CAD* coronary artery disease.

A total of 320 eligible subjects from the two centers were initially enrolled, among which 82 had been excluded, finally, 238 participants (including 128 patients with knee OA and 110 non-OA controls) finished the study and were included for analysis (flowchart as in Fig. 2). Their clinical characteristics are summarized in Table 1. A significant difference was found in body mass index (BMI), total cholesterol, low-density lipoprotein (LDL) cholesterol, triglycerides, and hs-CRP between two groups (all *P* < 0.05, Table 1).

**Table 1:** Clinical characteristics of the participants in this study.

	Total			Center 1			Center 2		
	OA Patients	Controls	*P* value	OA Patients	Controls	*P* value	OA Patients	Controls	*P* value
N	128	110		89	75		39	35	
Age (years)	61.69 ± 8.49	61.26 ± 7.868	0.692	62.18 ± 8.99	61.80 ± 8.33	0.781	60.56 ± 7.20	60.11 ± 6.73	0.783
Male/female (n)	42/86	37/73	0.893	28/61	26/49	0.663	14/25	11/24	0.685
BMI (Kg/m^2^)	25.77 ± 3.34	24.42 ± 3.29	0.002	25.62 ± 3.54	24.12 ± 3.67	0.009	26.10 ± 2.83	25.05 ± 2.20	0.081
Systolic BP (mmHg)	131 ± 12	128 ± 12	0.057	131 ± 13	128 ± 14	0.214	130 ± 10	126 ± 8	0.067
Diastolic BP (mmHg)	79 ± 7	78 ± 7	0.257	79 ± 7	77 ± 9	0.300	79 ± 7	78 ± 5	0.588
Mean BP (mmHg)	96 ± 8	94 ± 8	0.070	96 ± 8	94 ± 10	0.210	96 ± 7	94 ± 5	0.128
Hear rate (bpm)	71 ± 9	70 ± 8	0.294	71 ± 9	70 ± 8	0.435	71 ± 7	69 ± 8	0.477
FBG (mmol/L)	5.29 ± 0.77	5.18 ± 0.69	0.246	5.22 ± 0.76	5.16 ± 0.68	0.596	5.45 ± 0.76	5.22 ± 0.73	0.486
Total cholesterol (mmol/L)	5.49 ± 1.04	4.96 ± 0.82	< 0.0001	5.67 ± 1.10	4.94 ± 0.88	< 0.0001	5.07 ± 0.75	4.96 ± 0.69	0.491
HDL cholesterol (mmol/L)	1.20 ± 0.32	1.26 ± 0.36	0.199	1.20 ± 0.31	1.22 ± 0.37	0.612	1.21 ± 0.32	1.32 ± 0.33	0.249
LDL cholesterol (mmol/L)	3.41 ± 0.77	3.05 ± 0.78	0.001	3.55 ± 0.72	3.04 ± 0.76	< 0.0001	3.10 ± 0.80	3.05 ± 0.86	0.788
Triglycerides (mmol/L)	1.53 ± 0.74	1.27 ± 0.71	0.006	1.50 ± 0.65	1.23 ± 0.66	0.010	1.61 ± 0.93	1.35 ± 0.79	0.205
Smoking (n, %)	17 (13.3%)	13 (11.8%)	0.735	11 (12.4%)	9 (12.0%)	0.944	6 (15.4%)	4 (11.4%)	0.740
hs-CRP (mg/L)	1.78 ± 1.01	1.48 ± 0.80	0.014	1.73 ± 1.02	1.54 ± 0.81	0.206	1.89 ± 1.00	1.35 ± 0.79	0.012
Kellgren-Lawrence score	3(2, 4)	–	–	3(2, 4)	–	–	2(2, 3)	–	–
faPWV (m/s)	12.10 ± 2.09	11.67 ± 2.52	0.130	12.12 ± 1.88	11.69 ± 2.30	0.186	12.04 ± 2.53	11.63 ± 2.180	0.455
cfPWV (m/s)	9.40 ± 1.92	8.25 ± 1.26	< 0.0001	9.48 ± 2.03	8.23 ± 1.13	< 0.0001	9.23 ± 1.64	8.29 ± 1.52	< 0.0001

*BMI* body mass index, *BP* blood pressure, *FBG* fasting blood glucose, *HDL* high-density lipoprotein, *LDL* low-density lipoprotein, *hs-CRP* high-sensitivity C-reactive protein, *faPWV* femoral-ankle pulse wave velocity, *cfPWV* carotid-femoral pulse wave velocity.

**Figure 3:**
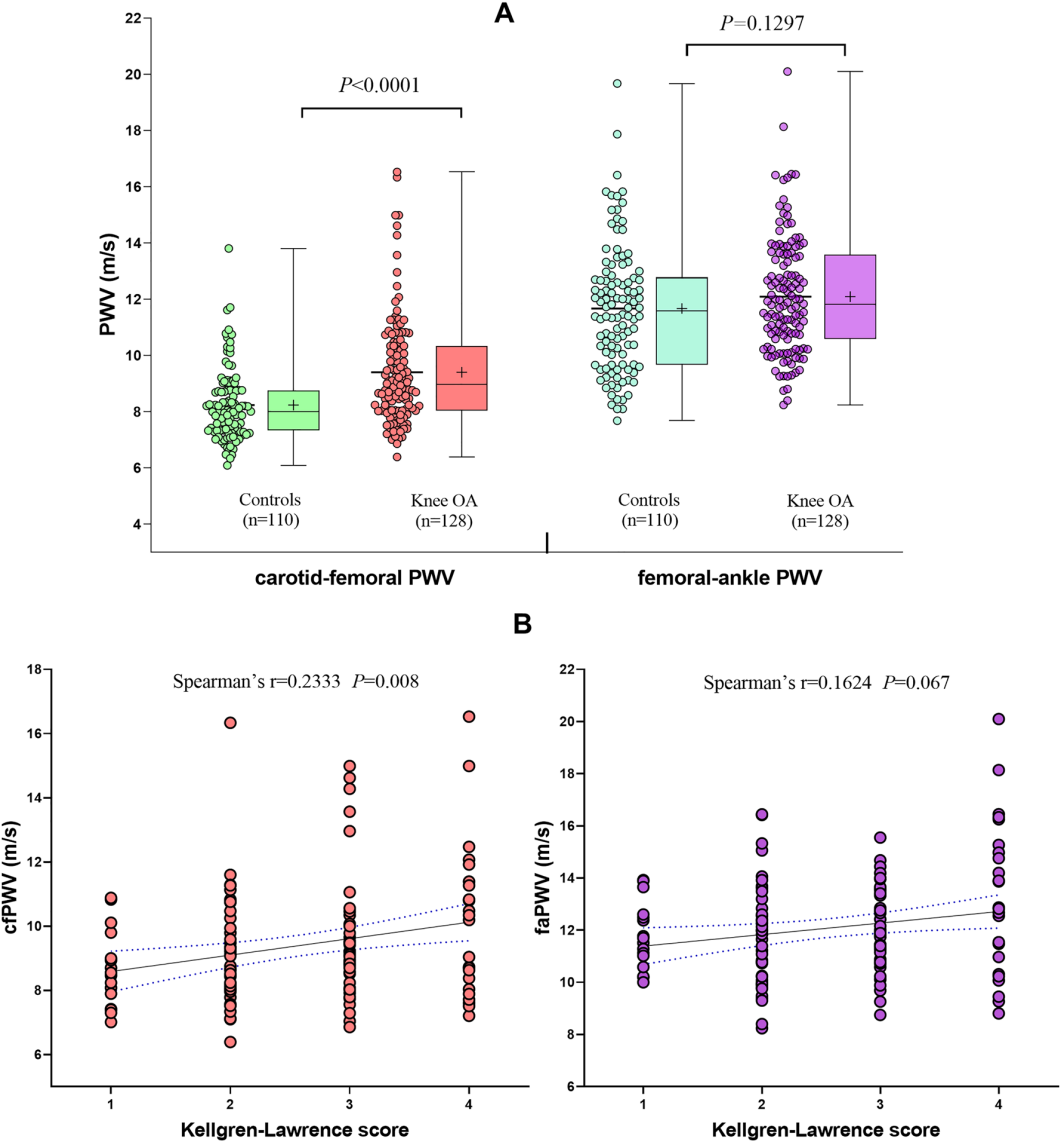
PWV measurements and correlation with Kellgren–Lawrence score. The cfPWV of patients with knee OA was significantly increased, but no significant difference of faPWV was found between two groups (**A**). Spearman’s correlation analysis showed that, in patients with knee OA, cfPWV but not faPWV, had a significant correlation with Kellgren–Lawrence score (**B**). *OA* osteoarthritis, *faPWV* femoral-ankle pulse wave velocity, *cfPWV* carotid-femoral pulse wave velocity.

In this study, the cfPWV measurements of patients with knee OA were significantly higher than that of non-OA controls (9.40 ± 1.92 *vs* 8.25 ± 1.26 m/s, *P* < 0.0001, Fig. 3A). However, there was no significant difference of faPWV measurements between two groups (12.10 ± 2.09 *vs* 11.67 ± 2.52 m/s, *P* = 0.130, Fig. 3A). In patients with knee OA, the Kellgren-Lawrence score was 3(2, 4). As shown in Fig. 3B, Spearman’s correlation analysis showed that cfPWV had a significant correlation with Kellgren-Lawrence score in OA patients (r_s_ = 0.2333, *P* = 0.008), in contrast, no correlation was founded between faPWV with Kellgren-Lawrence score (r_s_ = 0.1624, *P* = 0.067). Furthermore, after adjusting the confounders including age, gender, smoking, BMI, mean BP, LDL cholesterol and hs-CRP by multiple regression analysis, a significant correlation with Kellgren-Lawrence score and cfPWV still existed (*P* < 0.0001), but faPWV still showed no independent association with Kellgren-Lawrence score (*P* = 0.233).

Simple correlations between clinical characteristics and PWVs were investigated in this study and the results are demonstrated in Table 2. Two multiple regression models were used to determine the independent factors that may affect regional PWV of the total subjects included in this study. In model 1 (for cfPWV) and model 2 (for faPWV), age, gender, smoking, mean BP, LDL cholesterol, BMI and hs-CRP were routinely included, in addition, variables with *P* value < 0.20 in Table 2 were also added into each model.

**Table 2:** Univariate linear correlation analysis between factors with cfPWV and faPWV in total subjects.

	With cfPWV			With faPWV		
	Correlation coefficient (95% CI)	R^2^	*P* value	Correlation coefficient (95% CI)	R^2^	*P* value
Age	0.429 (0.319, 0.528)	0.184	< 0.0001	0.161 (0.035, 0.283)	0.026	0.013
BMI	0.075 (−0.053, 0.200)	0.006	0.250	−0.023 (−0.150, 0.105)	0.001	0.725
Mean BP	0.156 (0.029, 0.277)	0.024	0.016	0.173 (0.046, 0.293)	0.030	0.008
Heart rate	0.137 (0.010, 0.259)	0.019	0.035	0.054 (−0.073, 0.180)	0.003	0.405
FBG	0.062 (−0.066, 0.188)	0.004	0.341	−0.067 (−0.192, 0.061)	0.005	0.304
Total cholesterol	0.095 (−0.032, 0.220)	0.009	0.142	0.029 (−0.098, 0.156)	0.001	0.655
Triglycerides	0.196 (0.071, 0.316)	0.039	0.002	0.150 (0.023, 0.272)	0.023	0.021
HDL cholesterol	−0.126 (−0.249, 0.001)	0.016	0.052	−0.083 (−0.208, 0.044)	0.007	0.201
LDL cholesterol	0.066 (−0.062, 0.191)	0.004	0.312	0.026 (−0.102, 0.152)	0.001	0.694
hs-CRP	0.021(−0.154, 0.194)	0.000	0.817	0.018(−0.176, 0.156)	0.000	0.845
Gender	−0.031 (−0.162, 0.100)*		0.631	−0.030 (−0.160, 0.102) *		0.649
Smoking	−0.074 (−0.203, 0.057)*		0.255	−0.022 (−0.152, 0.109) *		0.737
Knee OA	0.355 (0.235, 0.464)*		< 0.0001	0.104 (−0.028, 0.231)*		0.111

R^2^, the coefficient of determination of Pearson’s correlation. Dummy variables were used for gender (female = 0, male = 1), smoking (current smoker = 1, non-smoker = 0), knee OA (yes = 1, no = 0). Abbreviations as in Table 1.

*Spearman’s correlation coefficient.

The results of the multiple regression analysis are displayed in Table 3. Model 1 included confounders including age, gender, smoking, BMI, mean BP, LDL and HDL cholesterol, total cholesterol, triglycerides, heart rate, hs-CRP, knee OA, and the results demonstrated that only age and presence of knee OA were independently associated with cfPWV (both *P* < 0.0001). While in model 2, confounders including age, gender, smoking, BMI, mean BP, LDL cholesterol, triglycerides, hs-CRP and knee OA were included, and the results showed that only age was independently associated with faPWV (*P* = 0.003), and no independent correlation between presence of knee OA and faPWV was founded (*P* = 0.372, Table 3).

**Table 3:** The analysis result of each multiple regression model in this study (n = 238).

	B (95% CI)	β	*P* value
Model 1 for cfPWV			
Constant	3.560 (0.092, 7.029)		0.044
Age	0.044 (0.025, 0.064)	0.275	< 0.0001
Gender	0.109 (−0.331, 0.549)	0.030	0.626
Smoking	−0.477 (−1.114, 0.160)	−0.091	0.141
BMI	−0.009 (−0.071, 0.053)	−0.017	0.776
Mean BP	0.016 (−0.009, 0.042)	0.076	0.212
LDL cholesterol	−0.075 (−0.321, 0.171)	−0.038	0.546
Total cholesterol	0.040 (−0.182, 0.261)	0.023	0.724
Triglycerides	0.188 (−0.113, 0.489)	0.079	0.220
HDL cholesterol	−0.257 (−0.874, 0.360)	−0.049	0.413
Heart rate	0.011 (−0.009, 0.030)	0.064	0.285
hs-CRP	0.074(−0.150, 0.297)	0.039	0.518
Knee OA	0.930 (0.481, 1.379)	0.267	< 0.0001
Model 2 for faPWV			
Constant	6.666 (2.608, 10.723)		0.001
Age	0.040 (0.014, 0.067)	0.201	0.003
Gender	−0.128 (v0.725, 0. 468)	−0.028	0.672
Smoking	−0.028 (−0.890, 0.833)	−0.004	0.948
BMI	−0.012 (−0.095, 0.072)	−0.018	0.782
Mean BP	0.032 (−0.004, 0.067)	0.117	0.078
LDL cholesterol	−0.053 (−0.379, 0.273)	−0.021	0.749
Triglycerides	0.301 (−0.090, 0.691)	0.102	0.691
hs-CRP	−0.164(−0.466,0.137)	−0.070	0.284
Knee OA	0.269 (−0.324, 0.861)	0.062	0.372

Model information: model 1, R = 0.493, R^2^ = 0.243, *P* < 0.0001, Durbin–Watson = 2.073; model 2, R = 0.298, R^2^ = 0.089, *P* = 0.01, Durbin–Watson = 2.048. B, unstandardized partial regression coefficient; β, standardized regression coefficient; dummy variables were used for gender (female = 0, male = 1), knee OA (yes = 1, no = 0). Abbreviations as in Table 1.

## Discussion

In this two-centered case–control study, for the first time, we measured and compared two regional PWVs, cfPWV (the “gold standard” measurement for aortic stiffness) and faPWV (reflecting the stiffness of lower limb peripheral arteries) in patients with primary knee OA and matched non-OA controls. Interestingly, the results showed that cfPWV, but not faPWV, was remarkably increased in patients with knee OA, suggesting that stiffening of aorta but not lower limb arteries more closely associated with knee OA. Our findings thus naturally linked OA with increased CVD risk through aortic stiffness, a robust independent predictor and contributor to CVD and mortality^5^.

The results of this study have supported our hypothesis and revealed that aortic stiffness may have more important implications in considering CVD risk in OA patients. It was reported that aortic stiffness measured by cfPWV enabled to improve prediction of CVD beyond conventional risk factors^14^. Theoretically, stiffening of aorta has more significant pathophysiology implications. In physiological condition, the elastic aorta exerts a powerful cushioning function, which limits arterial pulsatility and protects the microvasculature from potentially harmful fluctuations in pressure and blood flow. When aorta becomes stiffer due to aging and various pathologic states, the cushioning function can be impaired, resulting in important consequences including arterial hypertension, target organs injures (due to pulsatility penetrating into microcirculation), left ventricular remodeling, dysfunction, and even failure^15^. Importantly, the aortic stiffness increasing while the stiffness of peripheral arteries keeping unchanged, as found in this study, was called “impedance mismatch lost”^16^, which would decrease the pressure wave reflection and facilitate the blood flow pulsatility transmits into and damage the peripheral microcirculation (finally leading to tissue ischemia) which may be involved in the pathophysiology of knee OA. The direct ischemic effects on bone are known to reduce cartilage nutrition and inflict multiple bone infarcts that are characteristic of advanced OA^17^.

Our previous studies suggested that stiffness of different arterial segments in human body were unequally affected by rheumatoid arthritis^18^. The discrepant changes in stiffness between aorta and peripheral arteries found in this study may provide a new insight into understanding the pathogenesis of cardiovascular and microvascular dysfunction frequently occurred in chronic rheumatic diseases, including OA. On this basis, clinical implementation of cfPWV evaluation in OA patients, is thus expected to better understand the excess CVD risk and may help to extend future preventive management strategies beyond the current focus on treating chronic symptoms and surgery for advanced knee OA. The findings of this study may suggest that assessment of aortic stiffness (e.g., measuring cfPWV), rather than lower limb AS, is recommended to be preferably used in clinical practices for evaluating vascular pathology and CVD risk in patients with knee OA.

To date, very limited literature has been reported regarding AS in OA patients. Tootsi K et al. demonstrated that OA patients have increased cfPWV compared with non-OA control subjects^19,20^. In two echocardiographic studies, aortic elastic properties were observed decreased in patients with knee OA^21,22^, however, in these two studies, cfPWV (the “gold standard” parameter of aortic stiffness) was not used. Moreover, there was no any single study providing with the measurements and comparison of stiffness between aorta and peripheral arteries in knee OA before, where the technical challenge might be one of the reasons. A newly developed semi-automatic ultrasound system for cfPWV measurement used in this study made this issue became available. This new method was proved to be an accurate, simple and reproducible method^12^, and its operations can be easily grasped by clinical staff (even without ultrasound experience) after a very short-term training. These advantages may facilitate its utility in future routine practice, including in OA patients.

There were some potential limitations in this study. Firstly, this study was an observational, cross-sectional design, which only showed an association between aortic stiffness with knee OA and thus could not discover the causal relationship. The causal link or interrelationship between them still remains elusive and further evidences are needed. Secondly, non-OA controls included in this study did not have hand X-rays, some asymptomatic hand OA cases might be included and might contribute an unknown bias to the PWV measurements of the controls. Thirdly, in most subjects, data concerning physical activity was not collected but had been shown to influence AS. Therefore, physical activity is a confounding factor that was not accounted for in the present study. Another limitation was that, the measurement of PWV was performed using a novel ultrasound technique in this study. Given that probe-based cfPWV might be considered as the current “gold standard” method for assessment of aortic stiffness, as it has the most outcome data, the results using this novel ultrasound technique may need confirmation by other studies. Last but not the least, the findings of this study may suggest further implementation of aortic stiffness evaluation in OA patients. However, to what extent the OA patients would benefit from adding it into traditional assessing strategies should be carefully determined in future large-scale clinical studies.

## Conclusions

Taken together, this study demonstrated that stiffening of aorta, but not lower limb arteries, were independently associated with knee OA. The findings in this study may call for further implementation of aortic stiffness assessment in clinical practice to evaluate CVD risk in patients with OA.

## Data Availability

The datasets used and analyzed during the current study are available from the corresponding author on reasonable request.
